# Alternative Opportunistic Alert Diffusion to Support Infrastructure Failure during Disasters

**DOI:** 10.3390/s17102370

**Published:** 2017-10-17

**Authors:** Farouk Mezghani, Nathalie Mitton

**Affiliations:** Inria Lille—Nord Europe, 40 Avenue Halley, 59650 Villeneuve d’Ascq, France; nathalie.mitton@inria.fr

**Keywords:** disaster recovery, opportunistic communication, multi-network, energy consumption

## Abstract

Opportunistic communications present a promising solution for disaster network recovery in emergency situations such as hurricanes, earthquakes, and floods, where infrastructure might be destroyed. Some recent works in the literature have proposed opportunistic-based disaster recovery solutions, but they have omitted the consideration of mobile devices that come with different network technologies and various initial energy levels. This work presents COPE, an energy-aware **C**ooperative **OP**portunistic al**E**rt diffusion scheme for trapped survivors to use during disaster scenarios to report their position and ease their rescue operation. It aims to maintain mobile devices functional for as long as possible for maximum network coverage until reaching proximate rescuers. COPE deals with mobile devices that come with an assortment of networks and aims to perform systematic network interface selection. Furthermore, it considers mobile devices with various energy levels and allows low-energy nodes to hold their charge for longer time with the support of high-energy nodes. A proof-of-concept implementation has been performed to study the doability and efficiency of COPE, and to highlight the lessons learned.

## 1. Introduction

During disaster scenarios such as hurricanes, earthquakes, and floods, communication is needed for rescue operations of trapped survivors. However, network infrastructure might be damaged and thus may no longer be available, rendering mobile communication devices such as smartphones, tablets, and mobile phones practically useless. Opportunistic communications have been investigated as a promising method of communication after disaster events [1,2]. Indeed, although network infrastructure might be destroyed, mobile devices used daily by everyone (e.g., smartphones) are the most helpful communication tools, and can assist several disaster recovery services in several ways [3,4]. For instance, trapped survivors can use their mobile devices and communicate with rescuers using short-range communications to report their position, to make their rescue operation quicker and more efficient.

Several research works [3,4,5,6] proposed opportunistic-based disaster recovery schemes, although important features have been omitted. On the one hand, they did not consider mobile devices that come with multi-network assortment. However, mobile devices might use multiple network technologies (e.g., Wi-Fi, Bluetooth) and the choice of use is left to the user, who is unaware of what is the best, or may be in physical or psychological distress, preventing him/her from making this choice [7]. On the other hand, devices with various initial power levels, making low-energy nodes batteries drain quickly, have not been taken into account. This work aims to design an opportunistic alert diffusion scheme for a disaster recovery scenario that exploits the multiple network technologies available in mobile devices and takes various battery levels into account. This work presents COPE, an energy-aware **C**ooperative **OP**portunistic al**E**rt diffusion scheme, useful for trapped survivors during emergency situations. COPE aims to rapidly reach rescuers in close proximity, while maintaining survivor devices functional for as long as possible. COPE performance is evaluated based on a proof-of-concept study.

This paper proceeds as follows. Section 2 reviews the related works. Section 3 presents the system model and the COPE scheme. Section 4 details the proof-of-concept study and gives some evaluation results. Simulation results to evaluate COPE performance from the alert delivery success rate perspective are given in Section 5. Section 6 highlights lessons learned and gives future directions. Section 7 concludes this work and presents some future works.

## 2. Related Works

Several research works have proposed solutions that seek to improve disaster recovery, rescue operations, and emergency evacuation. Some works in the literature [5,6] evaluate the performance of reference routing algorithms such as Epidemic and MaxProp, and study their applicability to important tasks in disaster relief operations. The authors of [8] proposed a new technique that uses opportunistic routing to ensure reliable and continuous communication between the rescuers and other people during disaster scenarios. The authors of [9] proposed a post-disaster ad hoc communication architecture for collecting geolocalisation information about victims and survivors. The authors of [3,4] exploited opportunistic communication to collect messages in disaster scenarios and inform mobile users with emergency information such as that of impassable and congested roads to ease their evacuation. A multi-hop device-to-device communication scheme was proposed in [2] and uses smartphones to relay messages in the disaster area. The authors of [10] proposed cooperative alert diffusion, exploiting opportunistic communication and allowing for minimization of energy consumption. A recent study proposed a prototype that uses ad hoc networking smartphones of rescuers and civilians, as well as upgraded wireless home routers to enable different types of emergency services [11].

Even though many works in the literature have contributed to improving disaster recovery and rescue operations, some of the picture is still missing. The aforementioned works have considered mobile devices equipped with only one type of network technology. However, mobile devices might use multiple network technologies and the choice is usually left to the user, who is unaware of what is best, or may be in a physical or psychological distress, preventing him/her from making this choice. Furthermore, these works do not consider mobile devices that come with various energy levels.

This work focuses on opportunistic-based alert diffusion, which is useful for trapped survivors during disaster scenarios to ease and speed up rescue operations. It differs from previous studies in that it considers the assortment of networks and is based on an automatic and systematic network interface selection. Additionally, this work is based on cooperative diffusion and takes various energy levels into account. This allows for the preservation of battery power for as long as possible. Mobile devices with low battery levels are maintained functional for longer, as is the network coverage.

## 3. COPE: Cooperative Alert Diffusion Scheme

### 3.1. System Model

This work considers a set of nodes $S=\left\{S_{i}\right\}$, each one equipped with a mobile device. The latter is assumed to feature multiple network technologies $N=\left\{n_{j}\;|\;j\in \left[1\;\ldots N\right]\right\}$ and is characterized by a current battery power level $p_{S_{i}}$. The integrated network technologies are distinguished mainly by their energy consumption models *EC* and transmission ranges *TR*. This work assumes that network technologies can be ranked according to their transmission range *TR* and energy consumption *EC* such that:

$$\left\{\begin{array}{c} {\mathrm{TR}}_{n_{j+1}}>{\mathrm{TR}}_{n_{j}}\\ {\mathrm{EC}}_{n_{j+1}}>{\mathrm{EC}}_{n_{j}} \end{array}\right.$$

The different network technologies can be distinguished according to their transmission speeds. However, this feature is not considered of great importance since alert diffusion messages are supposed to have very short sizes, requiring a normal transmission speed. The alert message represents a short message that comprises mainly the node identifier and location information to ease the rescue operation.

We would like to emphasize that this model can also be suitable for a mobile network composed of nodes each having a single communication interface that can be managed by different transmission powers, consequently leading to different transmission ranges/energy consumptions. COPE dynamically copes with all kinds of devices and interfaces, making decision only on link characteristics, which makes it scalable and agile.

### 3.2. COPE Scheme

#### 3.2.1. Multi-Network and Various Energy Level-Based Cooperation

**Figure 1:**
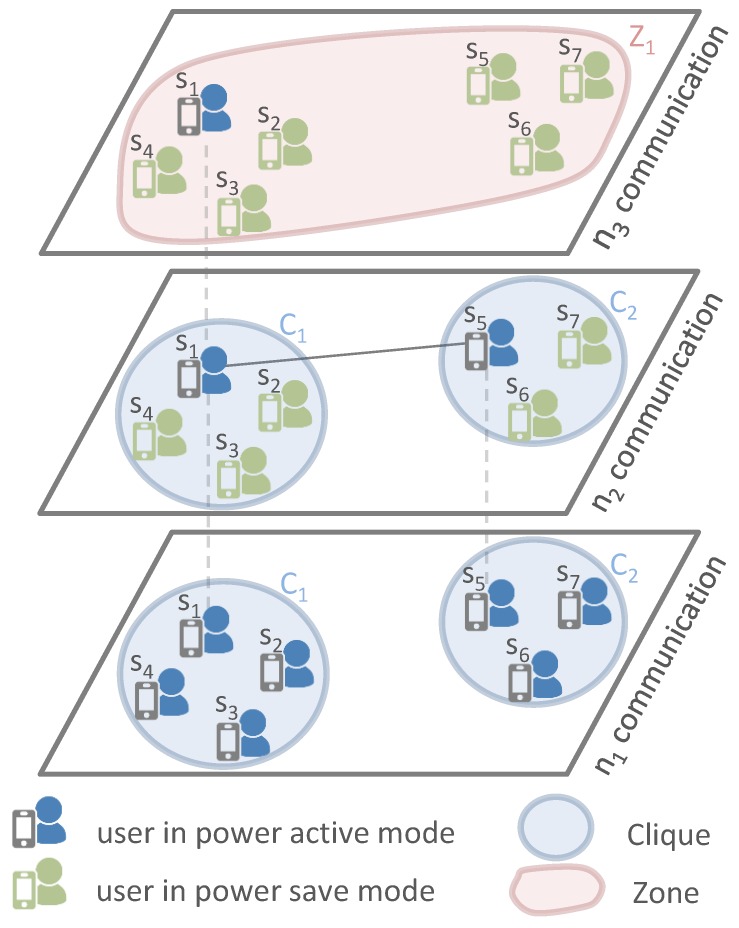
Layer communication overview.

In order to exploit the different network technologies while not draining the battery quickly, cooperative diffusion is considered in which nodes alternately diffuse the alert message. Moreover, to fit realistic conditions, COPE considers mobile devices that come with various energy levels. A power threshold $p_{th}$ is defined to distinguish low from high power nodes. With the support of high-energy nodes, COPE aims to maintain maximum network coverage for as long as possible by allowing low-energy nodes to stay alive for longer time. In the following, we describe a COPE scheme from the perspective of different network technologies, while Figure 1 illustrates a network technology-based layer communication view of COPE considering only three network technologies.

**Figure 2:**
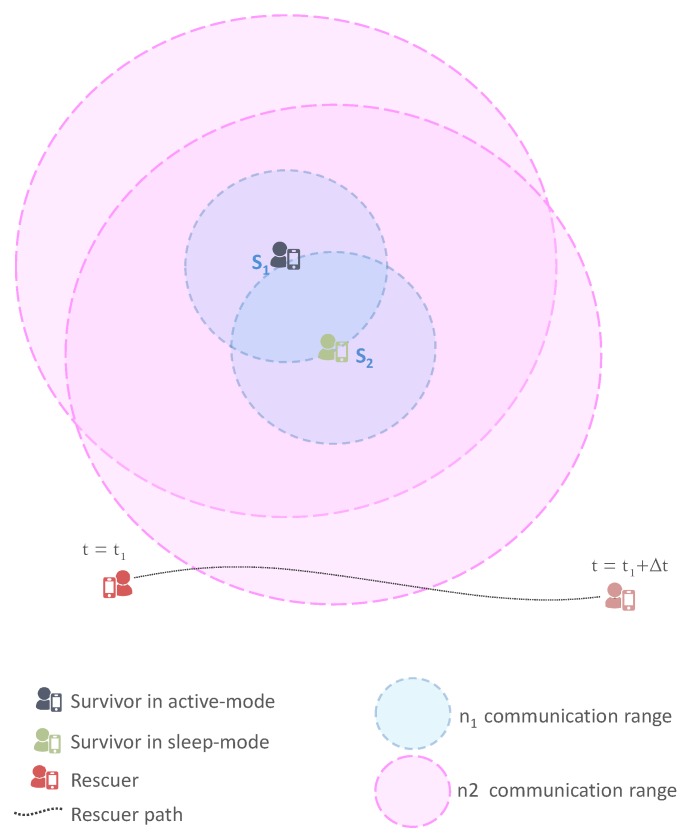
The particular case where the wake-up period of $S_{2}$ is too long: A rescuer node enters the area of the survivor node $S_{2}$ during its sleep period, but leaves it before it wakes up. The rescuer thus does not detect the survivor.

(1) *Layer $n_{1}$ Communication*: Nodes start discovering neighboring nodes for each interface and diffusing the alert message using lower power network technology $n_{1}$ ($n_{1}$ interface is maintained permanently active). The alert message represents a short text that mainly includes the position information of the trapped survivor to ease and speed up the rescue operation. Next, nodes diffuse their 1-hop $n_{1}$’s neighbor list, allowing for knowledge of 2-hop neighbors. Therefore, each node can determine the cliques to which it belongs for each interface. For instance, in Figure 1, node $S_{1}$ will discover it belongs to clique $C_{1}$ according to the network interface $n_{1}$.
(2) *Layer $n_{2}$ Communication*
  - *Periodic and cooperative diffusion*: Alternately, nodes discover proximate nodes and diffuse the alert message using $n_{2}$ network technology. Thus, the time horizon is divided into time slots (τ) and each time slot is divided into periods occupied by the nodes inside the clique. Indeed, each node computes its periodic wake-up schedule (i.e., compute the wake-up period and wake-up order during the time-slot) by referring to its energy level and identifier (ID) in comparison to those of nodes belonging to the same clique. First, each node computes its wake-up period (i.e., during which a node turns on its network technology for neighbor discovery and alert diffusion) considering the number of nodes inside its clique and by taking into account the fact that high-power nodes participate twice as much (double wake-up period) as compared to low-power nodes. For instance, let us assume that $S_{5}$ has a higher energy level than other nodes ($S_{6}$ and $S_{7}$) belonging to the same clique $C_{2}$ (e.g., $p_{S_{5}}>p_{S_{6}}+p_{th}$). Therefore, for $n_{2}$ alert diffusion, $S_{5}$ will have a wake-up period of 2τ/4, while the other nodes $S_{6}$ and $S_{7}$ will have a wake-up period equal to τ/4. Next, each node computes its wake-up order during τ based on its ID rank among those of other nodes inside the same clique (i.e., nodes having the lowest ID inside a clique occupy the first diffusion period). For instance, nodes $S_{1}$ and $S_{5}$ will wake up at the beginning of the time slot for interface $n_{2}$ since they have the lowest ID inside their corresponding cliques $C_{1}$ and $C_{2}$, respectively.
    We would like to emphasize that COPE considers the various energy levels and targets, with the support of high-power nodes, to maintain low-power nodes alive longer. Therefore, high-energy nodes have a longer wake-up period than low-energy nodes (participating for a greater amount of time in alert diffusion) to help the latter to preserve their batteries for a longer time. It would not be efficient to choose a very long wake-up period for high-power nodes as it would lead to a very high sleep period for low-power nodes. Nodes’ wake-up periods should be tuned to avoid the rescuer entering their coverage area without detecting it. Indeed, as illustrated in Figure 2, assuming the node $S_{1}$ has a long wake-up period and consequently $S_{2}$ has a long sleep period, a rescuer might enter the coverage of $S_{2}$ while it is in sleep mode and and leave it before it reaches wake-up mode. As a consequence, the rescuer would not receive the alert message. The optimal period has been studied in [12]. COPE ensures it is managed. Without loss of generality, we have simply assumed that high-power nodes participate twice as much as low-power nodes. We stress that nodes inside the same clique will have equal wake-up periods if their battery levels do not differ by more than $p_{th}$.
  - *Neighbor discovery*: If $n_{2}$ active nodes discover neighbors, they exchange information about their cliques (nodes belonging to the clique and their energy level) and they form a zone that includes their cliques. Then, they diffuse zone information (nodes belonging to the zone and their energy level) to their cliques through the active interface $n_{1}$. For instance, in Figure 1, nodes $S_{1}$ and $S_{5}$ will discover each other through the interface $n_{2}$. Hence, they exchange information about their corresponding cliques $C_{1}$ and $C_{2}$ and they form a new zone $Z_{1}$ comprising the two cliques $C_{1}$ and $C_{2}$. $S_{1}$ and $S_{5}$ diffuse the zone information to their cliques through interface $n_{1}$.
(3) *Layer $n_{3}$ communication*: Inside the formed zone, similarly each node computes its wake-up schedule by referring to its energy level and ID and to those of nodes inside the same zone. Then, nodes alternately diffuse the alert message and discover neighbors using the network technology $n_{3}$.
(4) *Layers $n_{j}$ & $n_{j+1}$*: Iteratively, from the $n_{j}^{th}$ communication perspective, nodes inside the same zone cooperate alternately to discover other proximate zones and alert potential proximity rescuers. If ever the active $n_{j}^{th}$ node discovers other nodes from another zone, they form a superior zone and inform other nodes belonging to the same clique/zone using the active interfaces. Then, cooperation inside the new zone is performed based on the network interface $n_{j+1}$.

#### 3.2.2. Topology Changes Due to Joining/Leaving Nodes

Nodes can detect joining or leaving nodes through the periodic messages. When this occurs, using the $n_{1}$ interface, nodes re-diffuse their 1-hop neighbors and update information about the cliques to which they belong. Receiving nodes will also update their own neighbor lists. Afterwards, this information is sent up to other $n_{j}$ layers through $n_{j}$ active nodes. Nodes accordingly update and re-compute their wake-up schedule. This work considers disaster scenarios whose network topologies are not changing that fast to make it better fit realistic conditions and to make the cooperation meaningful.

#### 3.2.3. Belonging to Multiple Cliques/Zones

**Figure 3:**
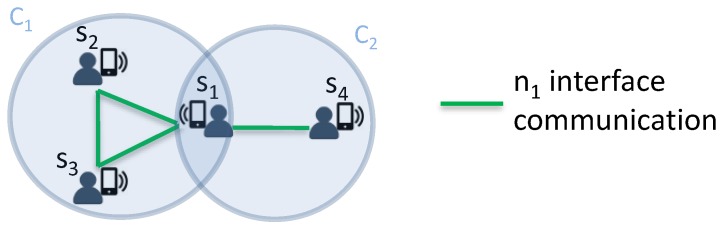
Example of node belonging to two cliques.

If a node belongs to more than one clique/zone, it computes its wake-up schedule considering the clique with the minimum number of nodes. Then, it informs nodes belonging to the same cliques/zones about its wake-up schedule so that it may be taken into consideration. By way of illustration, Figure 3 shows a simple example in which node $S_{1}$ is part of the cliques $C_{1}$ and $C_{2}$. Therefore, $S_{1}$ makes its wake-up schedule according to $C_{2}$ since it has fewer nodes than $C_{1}$. Assuming nodes with similar energy levels, $S_{1}$ will have a wake-up period of τ/2 and occupy the first half of the time slot since it has a lower ID compared to $S_{4}$. Afterwards, $S_{1}$ diffuses its wake-up schedule to its neighbors, allowing nodes $S_{2}$ and $S_{3}$ to take it into account to compute their wake-up schedules. Indeed, $S_{2}$ and $S_{3}$ will occupy the second half of the time slot for a wake-up period equal to τ/4.

#### 3.2.4. Emergency Alert and Rescuer Discovery

An emergency alert presents a short text message that mainly contains: (1) the node identifier; and (2) the location information of the survivor (e.g., GPS (Global Positioning System) coordinates). Survivors save the location information of other proximate trapped nodes belonging to other cliques. If a survivor gets a response, s/he automatically informs the rescuer about other proximate survivors and cliques to speed up rescue operations.

While it is not the focus of this work, an efficient design choice for size of different fields of the alert message is of a great importance since it impacts the alert diffusion scheme, mainly in terms of energy consumption. In this work, we have simply considered a short text message with the minimum required information to represent the alert message.

### 3.3. COPE Algorithm

Algorithm 1 gives the pseudocode for the COPE alert diffusion scheme run at each node. It describes how nodes cooperate and switch alternately between the active and sleep modes in a distributed way from the different network interface perspective.

First, nodes use the least powerful network interface for neighbor discovery and alert diffusion (Line 1). Neighbors form cliques and each node saves the information (i.e., neighbor ID and energy level) of its cliques, allowing for determination of the number of nodes inside a clique and computation of its rank among them (Lines 2–4). According to the energy level of the nodes and to ID ranks inside the clique, each node computes its wake-up period duration and its schedule during the time slot (i.e., diffusion start and end times) (Lines 5–7). Mobile devices are distinguished according to their batteries power and to the power threshold $p_{th}$. Then high-power nodes are considered to participate, in the alert diffusion, twice as much as low-power nodes (Lines 27–30). During its active mode (Line 10 i.e., the wake-up period), a node discovers neighboring nodes and diffuses the alert message based on the network interface $n_{j}$ (Line 11). Then, it forms a zone with neighbors from other cliques/zones and it sends the new zone information to other nodes belonging to the same cliques/zones using active interfaces (Lines 12–13). Each node can thus determine its wake-up schedule inside the formed zone according to its ID and energy level among those of other nodes inside the same zone (Lines 14–18). During the sleep mode, a node simply deactivates the network interface $n_{j}$ and preserves its energy (Lines 20–21). If a node gets an alert reply from rescuers, it sends the saved positions of proximate cliques and zones (Lines 24–26) in order to speed up the rescue operations.

**Algorithm 1** COPE—Run at each node.
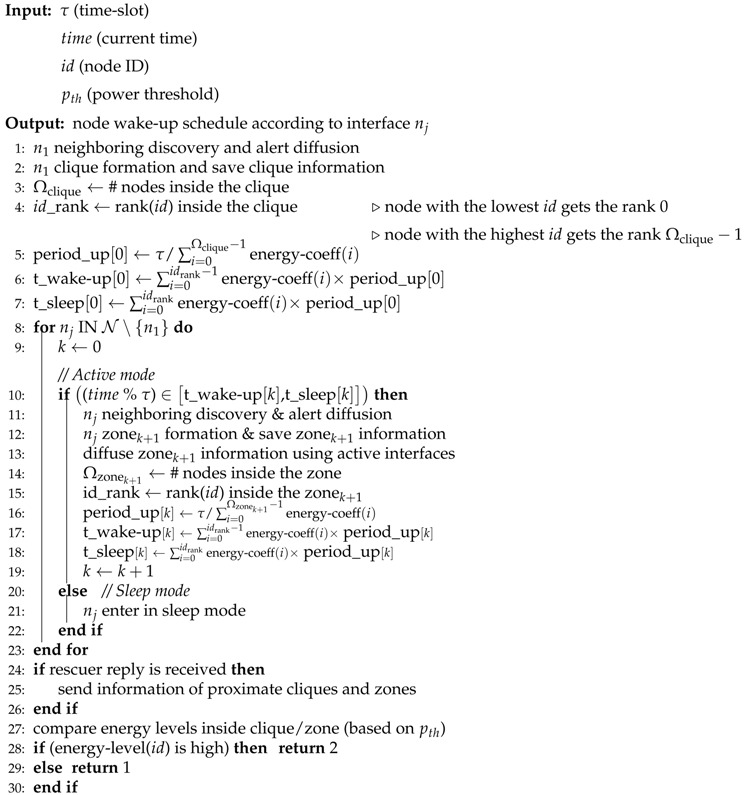


## 4. COPE: Proof-of-Concept

In the following, we detail the proof-of-concept carried out with smartphones using two network technology types: Bluetooth and WiFi. We would like to emphasize that COPE is technology-agnostic and works with as many communication technologies as available. Moreover, COPE can also be suitable for a mobile network composed of nodes that each have a single communication interface that can be managed by different transmission powers, consequently leading to different transmission ranges/energy consumption. COPE dynamically copes with all kinds of devices and interfaces, making decisions only on link characteristics.

This section focuses on the implementation of our opportunistic alert diffusion solution COPE in a real environment. Thus, a proof-of-concept study has been carried out to assess the doability and the functional validation of the proposed solution on the one hand and to point out the lessons learned on the other hand.

### 4.1. Experimentation Environment

The testing environment involves two main elements: mobile devices useful for opportunistic communication, and a DTN (Delay Tolerant Network) Bundle Protocol platform useful for neighbor discovery and alert diffusion.

#### 4.1.1. Mobile Devices: Communication Technology and Energy Level

**Figure 4:**
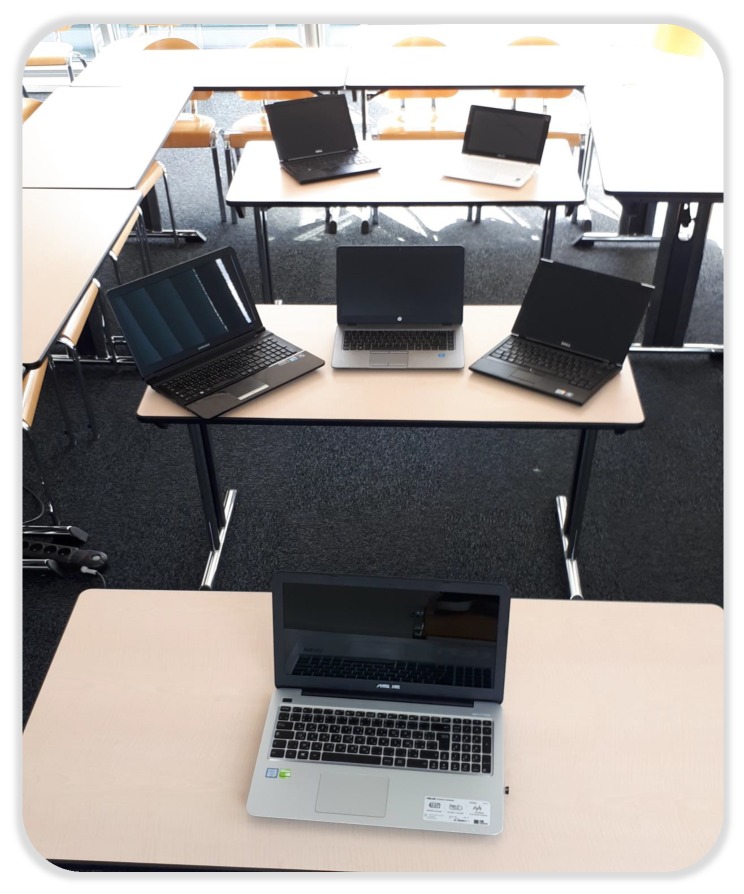
Linux laptops.

Figure 4 presents the testing environment, that involves six laptops, each equipped with the short-range communication technology types Bluetooth and Wi-Fi. The latter is distinguished according to communication power consumption and to transmission range. Indeed, Bluetooth consumes less energy, while offering a lower transmission range than Wi-Fi technology.

As a considerable amount of time is required to determine the power consumption of the different devices, the power level has been emulated and expressed in terms of energy units. The initial energy level of the nodes is in the range of $\left[0,N_{u}\right]$, where $N_{u}\in N$. The power threshold $p_{th}$ is set to 200 energy units during the testing environment to differentiate low from high-power nodes. We stress that $p_{th}$ has an impact on the battery lifespan of nodes presenting various energy levels and belonging to the same clique. Indeed, high-power nodes stop supporting low-power nodes when the difference of power levels is less than $p_{th}$. Hence, a small value of $p_{th}$ consists in supporting low-power nodes until reaching an energy balance (i.e., energy levels of nodes inside the same clique do not differ by more than $p_{th}$). Therefore, to make nodes batteries stay alive for similar periods of time, it is worth considering a small value of $p_{th}$, referring to the energy levels of the nodes. A high value of $p_{th}$ is equivalent to equity-based diffusion, which leads to a battery drain of some nodes much earlier than others. Some works in the literature have compared power consumption considering wireless network technologies such as Wi-Fi and Bluetooth [13]. Comparison results have shown Wi-Fi communication consumes more energy than Bluetooth communications. This work assumes that Wi-Fi transmissions consume three times the energy of Bluetooth transmissions.

Table 1 presents the characteristics of the different devices. The experimentation environment considers the physical MAC (Media Access Control) address of each device as a unique node identifier. For the sake of readability, integer values [1, 5] have been assigned to rank the different IDs of the survivors. For instance, as depicted in Table 1, node $S_{1}$ represents the node with the lowest MAC address compared to those of other survivor nodes.

#### 4.1.2. COPE Application and DTN Bundle Protocol

The proposed cooperative alert diffusion scheme, COPE, is implemented in C, C++ based on Linux and is accomplished by DTN2 [14], a reference implementation of the DTN bundle protocol defined in Request for Comments RFC-5050 [15], and offers different types of discovery agents. DTN2 supports different convergence layers such as TCP (Transmission Control Protocol), UDP (User Datagram Protocol), Bluetooth, and Ethernet convergence layers. It also includes support for several link types such as always on links, on-demand links, and opportunistic and scheduled links. The COPE application uses DTN2 as an experimental platform for opportunistic Bluetooth and Wi-Fi discoveries and communications. WiFi is configured in ad hoc mode for the COPE application. The basic workflow of DTN2 on Linux machines is as follows. Convergence layers need to be configured, which are used to transmit messages between machines. We have considered two convergence layers for the two network technologies used during the experimentation (i.e., Wi-Fi and Bluetooth). We have used the corresponding convergence layer for each type of network technology. Indeed, the TCP convergence layer has been used for the Wi-Fi communications, while the Bluetooth convergence layer has been used for the Bluetooth communications. Next, the nodes listen for neighbors beacons and distribute neighbor discovery events to the convergence layer. In the meanwhile, the service discovery announces the convergence layer’s availability to neighbors. Opportunistic links are then created with detected neighbors. Afterwards, message transmission with neighbors is performed based on the COPE algorithm.

#### 4.1.3. Proof-of-Concept Scenario

**Figure 5:**
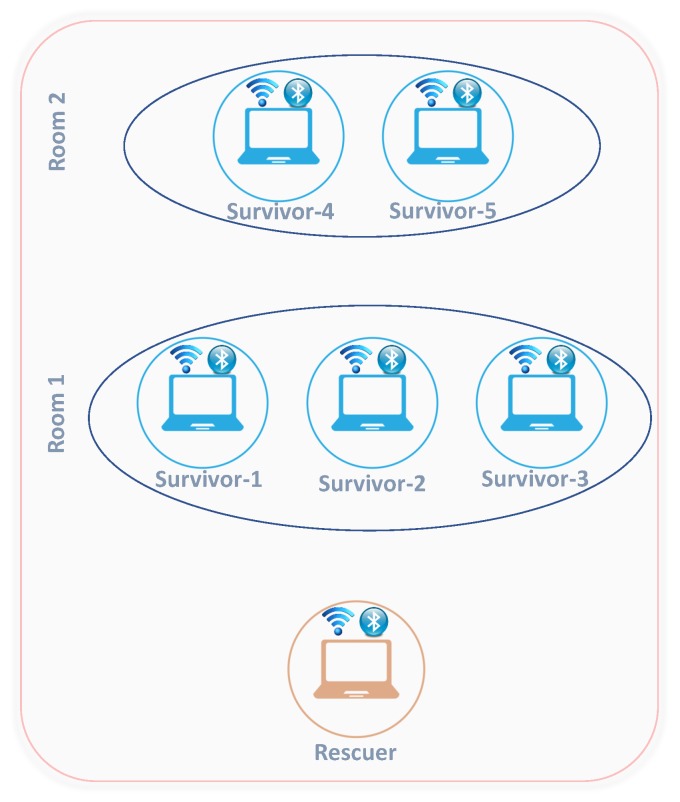
Proof-of-concept topology.

The adopted testing environment comprises five survivor nodes and one rescuer node. Survivors are separated into two distant rooms (Room 1 and Room 2) to obtain the topology as illustrated in Figure 5. Considering this topology, nodes inside the same room can communicate using Bluetooth and WiFi interfaces. On the other side, due to the short transmission range of Bluetooth, only WiFi communication can be established between nodes belonging to different rooms (Room 1 and Room 2). Finally, the rescuer node, considered to have no energy constraint, is initiated during the experimentation scenario and moved into the WiFi coverage area of the survivors to receive the alert message.

### 4.2. COPE Implementation

COPE implementation was performed based on the two available technology types Bluetooth and Wi-Fi. Nodes periodically exchange short messages of 4 s, mainly containing their ID and energy level. In order to make the energy consumption fit realistic cases more closely, we would like to emphasize that the energy level is computed considering heterogeneous mobile devices and heterogeneous batteries with different levels of energy consumption.

When Bluetooth neighbors are discovered, nodes exchange their 1-hop Bluetooth’s neighbors list allowing the knowledge of 2-hop neighbors in order to form cliques inside which they cooperate based on the Wi-Fi technology. Indeed, knowing their neighbor’s IDs and energy-levels, each node can determine the wake-up period during which it activates its Wi-Fi and diffuses the alert message, otherwise it turns its Wi-Fi into sleep mode.

After Bluetooth neighbor discovery, nodes inside Room 1 (Room 2, respectively) form a clique comprising nodes 1, 2, and 3 (4 and 5, respectively) as shown in Figure 5. Afterwards, Wi-Fi based cooperative alert diffusion is performed inside the formed cliques. According to node energy levels (see Table 1), $S_{1}$ ($S_{5}$ respectively) has a high power level compared to its cliques’ neighbors $S_{2}$ and $S_{3}$ ($S_{4}$ respectively).

**Figure 6:**
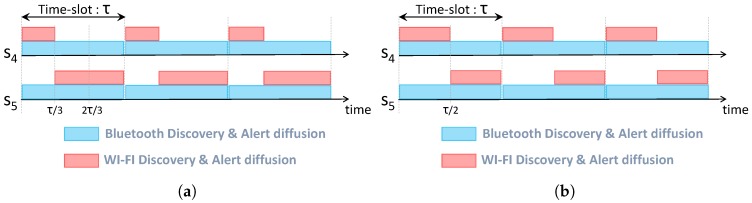
Alert diffusion schedule inside Room 2. (**a**) Before energy balance; (**b**) After energy balance.

**Figure 7:**
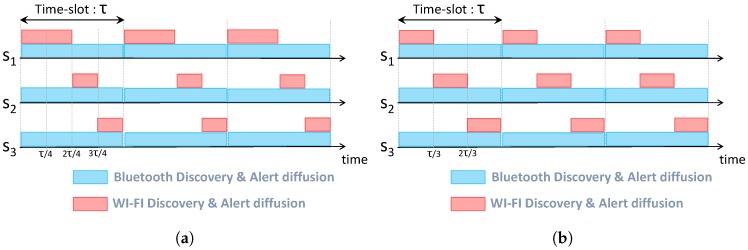
Alert diffusion schedule inside Room 1. (**a**) Before energy balance; (**b**) After energy balance.

Figure 6 and Figure 7 illustrate the wake-up schedules of survivors located in Room 1 and Room 2, respectively. As depicted in Figure 6a and Figure 7a, high-energy nodes ($S_{1}$ from Room 1 and $S_{5}$ from Room 2) have a wake-up period twice longer than low-energy nodes. Indeed, $S_{1}$ has a wake-up period of 2τ/4 while it is equal to τ/4 for $S_{2}$ and $S_{3}$. Similarly, $S_{5}$ has a wake-up period of 2τ/3, whereas it is τ/3 for $S_{4}$. After making an energy balance inside the clique, nodes cooperate based on equal wake-up periods (τ/3 for nodes inside Room 1 and τ/2 for nodes inside Room 2) as depicted in Figure 6b and Figure 7b.

### 4.3. Evaluations

**Figure 8:**
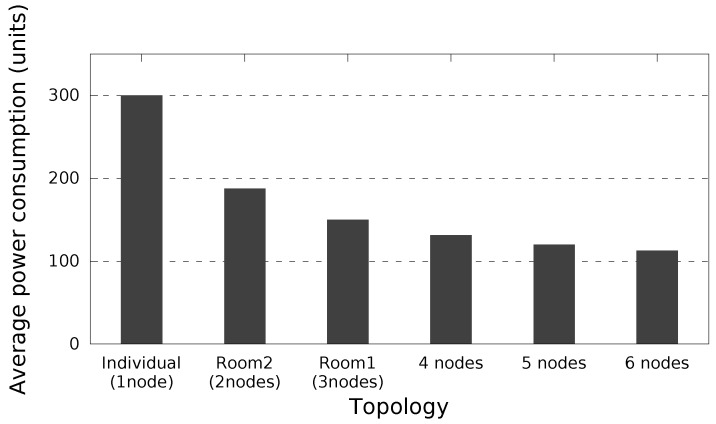
Average power consumption considering different topologies.

Figure 8 shows the average power consumption over 5 min considering different topologies: one node awake all the time (individual diffusion scheme), and a group of up to six nodes cooperating based on COPE. As can be seen from Figure 8, COPE allows survivors to save a significant amount of battery power compared to the individual diffusion scheme. Furthermore, the number of nodes increases as the power consumption is reduced, allowing survivors’ devices to stay functional for a longer time. The proof-of-concept scenario allows nodes to save energy from the Wi-Fi communication perspective. Obviously, considering testing environments with more network technologies will significantly decrease the energy consumption compared to the individual alert diffusion.

**Figure 9:**
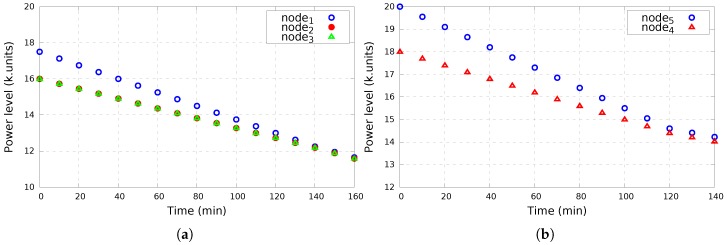
Power consumption per node. (**a**) Power consumption per node inside Room 1; (**b**) Power consumption per node inside Room 2.

COPE considers devices that initially come with various energy levels (as shown in Table 1) and allows low-energy nodes to hold their charge for longer time with the support of high-energy nodes. Figure 9 presents the power level of each node over time of each node inside Room 1 (Figure 9a) and Room 2 (Figure 9b), respectively. It is shown that during the time and with the support of high-energy nodes, an energy balance is established between nodes inside the same clique allowing low-energy nodes to maintain for longer time and so the network/survivor coverage.

We would like to emphasize that even though the experimentation environment has considered static nodes inside different rooms, some scenarios have been conducted to verify the topology dynamicity. Indeed, we have carried out different scenarios during which some nodes leave and join a clique. Based on the periodic exchange between nodes belonging to the same clique, new information about leaving/joining nodes is exchanged allowing each node to update and compute its wake-up schedule.

To endorse the previous results, the following section evaluates, through simulations, the performance of COPE in terms of alert delivery success ratio considering a dynamic disaster environment. Indeed, an efficient alert diffusion scheme needs to maintain mobile devices functional as long as possible while guaranteeing the delivery of emergency alerts to the potential rescuers. Thus, the alert delivery success rate has been evaluated through a disaster environment considering mobile survivors and a rescuer-node with various paths and velocities.

## 5. Alert Delivery Efficiency

The alert delivery success rate has been evaluated through simulations conducted using the Opportunistic Network Environment (ONE) [16]. Simulation scenarios involve a number of 35 mobile users considered as survivors. The mobility generator of BonnMotion has been used to generate mobility traces of users in a disaster scenario [17]. The BonnMotion disaster mobility model generates movements driven by tactical reasons based on a method called “separation of rooms” [18]. Using this method, the disaster scenario is divided into different context-based areas which are: the incident site, casualty treatment area, transport zone, and technical operational command zone. The simulation environment considers a disaster area comprising seven incident locations (e.g., buildings, parking, restaurant) in which trapped survivors are randomly distributed waiting for help.

Survivors are considered equipped each with a mobile device (e.g., smartphone) with two network interfaces corresponding to Bluetooth and Wi-Fi technologies and an initial energy level chosen randomly in the range of [10 k, 20 k] energy units.

Conducted simulations consider 100 scenarios involving a rescuer node that moves with a random path inside and around the disaster area combined with four different velocity ranges (1–1.5 m/s, 2–4 m/s, 6–8 m/s, and 12–14 m/s). We compute successful emergency alerts that have reached the rescuer and compare COPE, Selfish, and Equality-based alert diffusion. Selfish diffusion considers that each survivor only counts on himself for his survival. Equality operates similarly to COPE but does not consider the various energy levels. Indeed, it consists in a cooperation for equal periods of time independently from the various energy levels between nodes. First simulation scenarios compare the different alert diffusion schemes (Selfish, Equality, and COPE) with respect to the energy consumption and the alert delivery success ratio metrics.

**Figure 10:**
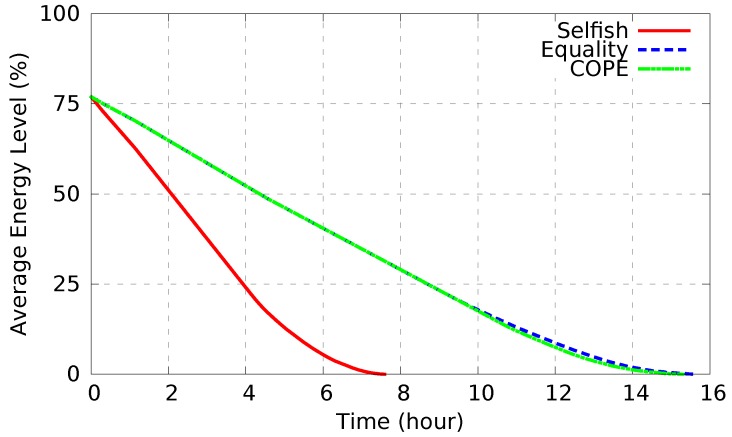
Average energy level over time.

Figure 10 shows the average energy level over time. Selfish diffusion results in a quick battery drain (average lifetime of 7.5 h). However, rescue operations might take long time. On the contrary, cooperative-based diffusion schemes significantly increase the battery lifetime for more than 14 h.

For a fair comparison with selfish diffusion, we first conducted scenarios considering the period of time [0, 1.5 h] (see Figure 10) during which all nodes are still alive (i.e., still have power in their batteries). To compute the alert delivery success rate, we ran each experiment 30 times using various rescuer paths at random intervention times in the range of [0, 1.5 h], and the results represent the average values within a 95% confidence interval.

**Figure 11:**
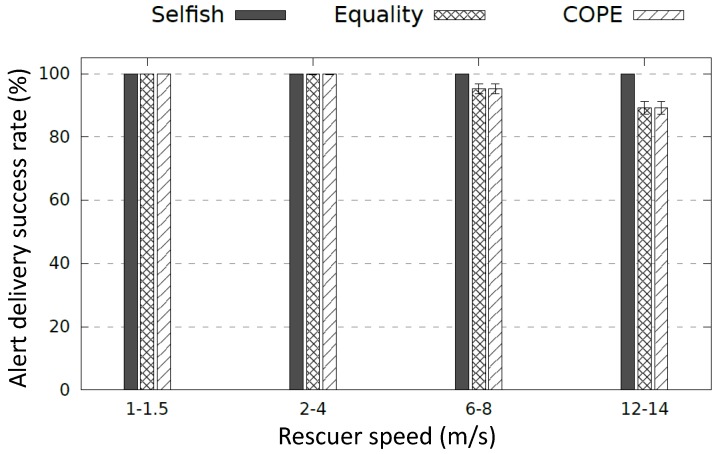
Alert delivery success rate where all nodes are alive.

As depicted in Figure 11, the different diffusion schemes succeed to reach the rescuer with walking and running speed (1–1.5 m/s and 2–4 m/s). When the rescuer velocity increases (6–8 m/s and 12–14 m/s, respectively), the alert delivery ratio decreases considering cooperative alert diffusion methods COPE and Equality. Indeed, a rescuer node can enter and leave the coverage of a Wi-Fi sleep node before its wake-up. Even though a high-speed rescuer is not realistic during a disaster, cooperative diffusion methods can manage this situation by reducing the time-slot enabling a fast switching between sleep and active modes. A further study to determine the efficient time slot size for the cooperative alert diffusion scheme is a focus of our future work. On the one hand, a short time slot may affect cliques comprising a high number of nodes. Considering a short time slot (e.g., 10 s) and a clique of a high number of nodes (e.g., 10 nodes), the nodes’ wake-up time is too short (∼1 s) according to COPE. However, in practice, a short wake-up period might be not enough to discover neighbors and diffuse the alert message. On the other hand, a long time slot may impact cliques with a small number of nodes since a rescuer might enter and leave the coverage of a sleep node before its wake-up.

**Figure 12:**
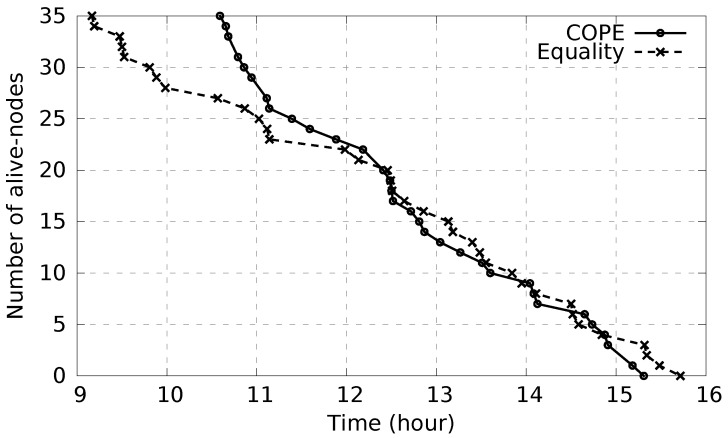
Network node disappearance due to lack of battery.

To show the impact of the various energy levels on the alert delivery success rate, in the following we compare COPE and Equality diffusion schemes based on a period of time during which nodes’ batteries start running out of power. COPE considers the various energy levels between nodes and aims, with the support of high-power nodes, to maintain the battery lifetime of low-power nodes for as long as possible. Indeed, as shown in Figure 12, considering COPE, node batteries start to get empty after 10.5 h. In contrast, considering equality-based diffusion, batteries of low-power nodes start draining after 9 h. Therefore, considering the various energy levels, COPE maintains large network coverage by maintaining the maximum number of nodes alive as long as possible. This leads to the waste of a few minutes from the battery lifespan of high-power nodes, allowing low-power nodes to gain a few hours of battery lifetime, consequently maintaining large network coverage for longer time.

**Figure 13:**
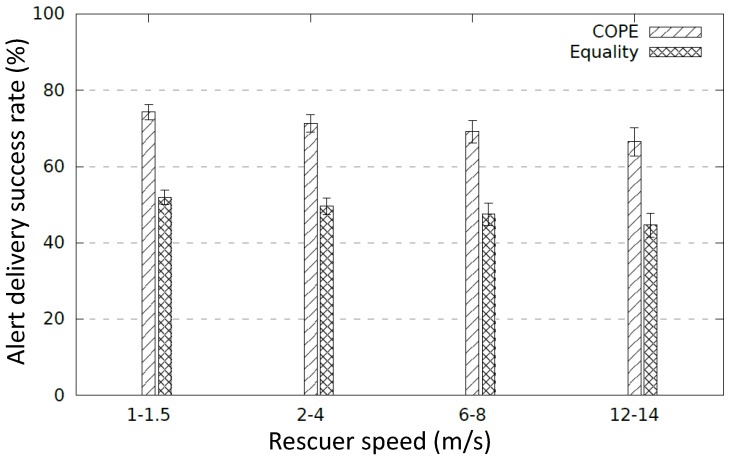
Alert delivery success rate where some nodes are still alive.

Simulations have been conducted to measure the alert delivery success rate during the period of time [9 h, 13 h] (see Figure 12) where only some nodes have energy left. To compute the alert delivery success rate, we have considered a rescuer node that moves with various paths (100 random paths) inside the disaster area at random intervention times during the period [9 h, 13 h] and we compute the number of successful emergency alert messages that have reached the rescuer. We ran each experiment 30 times and the results represent the average values within a 95% confidence interval. Figure 13 shows that COPE clearly outperforms Equality with respect to the alert delivery success ratio. This is because COPE allows maximum network coverage for a longer time by considering the various energy levels and thus allowing low-power nodes to stay alive longer.

## 6. Discussion and Future Directions

### 6.1. Node Synchronization

- This work assumes that nodes are already synchronized since mobile devices obtain the local time from the network providers with millisecond accuracy before disasters occur. Moreover, as the time-slot τ is at second level, no additional synchronization is required. Indeed, existing works in the literature [19] have tested the clock drift on different smartphones and results have shown that the clock skew is around one to two seconds per day, which is convenient for our system model since a rescue operation might take few hours. However, if nodes can lose synchronization over time and a time-shift occurs between nodes clock, the system can be adapted by adding further exchange, allowing distributed synchronization.
- Due to node desynchronization, a node can misbehave when evaluating its wake-up period and those of others belonging to the same clique. For instance, if the energy difference between two neighboring nodes $S_{1}$ and $S_{2}$ become under the power threshold $p_{th}$ while node $S_{1}$ still has an information that this energy difference is over $p_{th}$, in this case, a small period of time (during the time-slot τ) could not be covered by any nodes belonging to the same clique or in another case a small period of time could be covered by more than 1 node. However, these cases can occur very rarely and this would not affect the general functioning of the COPE scheme since information retrieved from the periodic messages would rapidly correct the wake-up schedule. Indeed, if a node misbehaves, through the wake-up schedule evaluation considering information retrieved from the previous periodic messages, it would correct its wake-up schedule when receiving the next neighbor messages. It would only impact one time period.

### 6.2. Survivor Wake-Up Order

**Figure 14:**
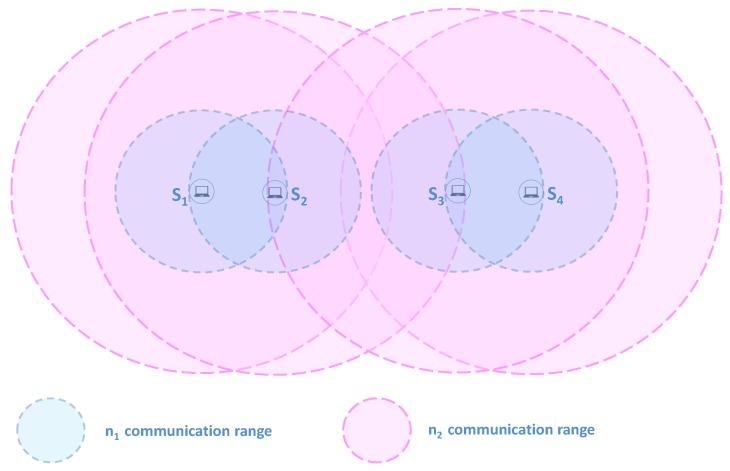
Example of four nodes.

Even though the wake-up order during the time slot is not the main focus of this work, a deep study of this latter is required. Indeed, efficient ordering can be performed considering other important criteria such as node positions. For instance, Figure 14 illustrates a sample scenario involving four nodes equipped each with multiple network interfaces. Assume that nodes have equal energy and that $S_{1}$ and $S_{2}$ ($S_{3}$ and $S_{4}$ respectively) can communicate and can thus form a clique based on the $n_{1}$ interface. Therefore, according to the node ID, $S_{1}$ ($S_{3}$, respectively) will be in wake-up mode during the first half of the time slot, while $S_{2}$ ($S_{4}$ respectively) will be in wake-up mode during the second half of the time slot for $n_{2}$ neighboring discovery and alert diffusion. However, communication between cliques using $n_{2}$ can no longer happen since only $S_{2}$ and $S_{3}$ are within range. Therefore, we can adaptively aggregate different criteria in the node wake-up order algorithm.

### 6.3. Network Technology Limitations

We would like to emphasize that COPE is suitable to network environments involving as many communication technologies as available. However, in practice, communication technologies such as Bluetooth might present some limitations. Indeed, using Bluetooth, each node can communicate with up to seven neighbors.

### 6.4. Network Dynamicity and Periodic Message Frequency

When a node leaves/joins a new clique, a rapid information update is required, allowing each concerned node to recompute its wake-up according to the different network interfaces. Hence, it is important to study the periodic message frequency and optimize it to fit the network mobility and to study its impact on the network overhead and energy consumption.

### 6.5. Heterogeneous Nodes and Available Communication Interfaces

COPE considers mobile devices equipped with the same network technologies. However, available communication interfaces might differ from a node to another. Hence, future works should adapt this solution to a network environment that comprises heterogeneous mobile devices in terms of network technologies and their number.

## 7. Conclusions

This work investigates the alert diffusion in disaster scenarios. It proposes COPE, a novel cooperative alert diffusion scheme that leverages multiple network technologies integrated in mobile devices and takes various energy levels into account. A proof-of-concept implementation of COPE has been carried out, based on laptops equipped with two short-range forms of communication (Wi-Fi and Bluetooth), and has shown the doability of COPE and its efficiency in terms of energy consumption. Adding another form of network technology to the proof-of-concept implementation is a subject of our ongoing work. The investigation of a disaster scenario from the rescue operation side will be the focus of our future work. For instance, determining the number of rescuers and the best rescuer path are important for speeding up rescue operations.

## Figures and Tables

**Table 1:** Node characteristics.

Location	Node ID (@MAC)	Initial Energy (Units)	Linux OS	Wi-Fi	Bluetooth
Room 1	$S_{1}$	17.5 k	Ubuntu 14.04	IEEE 802.11 a/b/g/n	4.0
	$S_{2}$	16 k	Ubuntu 14.04	IEEE 802.11 a/b/g/n	2.1
	$S_{3}$	16 k	Ubuntu 14.04	IEEE 802.11 a/b/g	3.0
Room 2	$S_{4}$	18 k	Ubuntu 14.04	IEEE 802.11 a/b/g/n	4.0
	$S_{5}$	20 k	Ubuntu 14.04	IEEE 802.11 a/b/g	2.1
	$R_{1}$	no energy constraint	Ubuntu 14.04	IEEE 802.11 a/b/g/n	4.0
